# Social categorization based on permanent versus transient visual traits in neurotypical children and children with autism spectrum disorder

**DOI:** 10.1038/s41598-021-85924-w

**Published:** 2021-03-22

**Authors:** Orsolya Kiss, Katalin Oláh, Lili Julia Fehér, József Topál

**Affiliations:** 1 Institute of Cognitive Neuroscience and Psychology, Research Centre for Natural Sciences, 2 Magyar Tudósok krt, Budapest, Hungary; 2 MTA-ELTE Social Minds Research Group, Eötvös Loránd University, 46 Izabella u., Budapest, Hungary; 3 Pázmány Péter Catholic University, 1 Mikszáth Kálmán tér, Budapest, Hungary; 4 Department of Cognitive Science, Budapest University of Technology and Economics, 1 Egry József U., Budapest, Hungary; 5 Center for Health Sciences, SRI International, Menlo Park, CA USA

**Keywords:** Human behaviour, Psychology

## Abstract

The present study was designed to test the relative weight of different types of category markers in children’s representations of social and biological kinds. We reasoned that in order to efficiently navigate through the mesh network of overlapping social categories, the representational system dedicated to processing information about social groups should be prepared to flexibly switch between potential ways of categorizing fellow humans. Thus, we hypothesized that children would assign more relevance to transient but symbolic features, such as shirt colour, when categorizing humans than other animal species. Across two experiments, we investigated whether typically developing children as well as children diagnosed with Autism Spectrum Disorder would categorize drawings of humans and dogs along a transient or a biologically set, permanent marker. The results show that both groups of children overwhelmingly selected the permanent feature to categorize dogs, however, they were more likely to categorize fellow humans based on transient features. We suggest that this tendency lays the ground for humans’ ability to efficiently represent the complex structure of societies.

## Introduction

One key human cognitive capacity that underlies the success of complex interactions is our tendency to structure information about fellow humans in terms of *social categories*. Social categorization in adults has been extensively investigated both in relation to the cognitive process of categorization itself and its (affective) implications for social interactions. For example, it is a well-documented phenomenon that humans have a tendency to categorize each other based on any arbitrary distinction (’minimal group’) that is made salient in a given context and that this distinction will immediately result in a preference for people belonging to the same group as oneself^1^. Nonetheless, some category distinctions have been suggested to take precedence over others: namely, age, gender and race have been typically considered to be encoded automatically upon encountering someone^2^. These distinctions have also been repeatedly described to form the grounds of robust stereotypes and prejudice^3^. However, the idea that all of these category dimensions would carry the same relevance for human cognition has been contested. For example, it has been suggested that the apparently automatic encoding of race may simply be a by-product of other evolutionary adaptive cognitive mechanisms that have developed in order for humans to efficiently map out coalitional relations within the society^4–6^.

The importance of social categorization in cognition is further supported by a body of research with young children, suggesting that humans may have a strong tendency to process social information in terms of categories^7–9^. Looking time studies, for example, have revealed that even preverbal infants prefer to look at “in-group” members based on sex^10^, race^11^ and language use^12^. Moreover, linguistic group membership guides social learning processes from the age of 14 months^13–16^. Importantly, it has been argued that these preferences are underlain by an early emerging system for social categorization that bears the same features as categorization in adulthood^9^. This claim is supported by findings that young children not only show preferences based on certain traits corresponding to social groups but also make inferences about the relationship of people based on perceived similarities in these features^9,17,18^. In addition, 3-year-olds also raise different expectation towards in-and out-group members in adhering to social norms: social norm transgressions are judged more severely when committed by an in-group member^19^.

Together, such findings have led researchers to formulate theories arguing that the human mind is equipped with a cognitive module that has evolved to efficiently guide information processing about the social environment^7,8^. However, the operation of this cognitive module is still unclear in many respects. As category representations in general, social categories are supposed to help us quickly acquire generic knowledge about kinds and to use these efficiently in inferences about the behaviour of individuals. Category representations are thus effective if they can grasp stable and generic characteristics of the kind that will yield correct predictions about the properties and behaviour of the members of the category. This is reflected in a specific feature of the human mind, called psychological essentialism^20^. The term refers to humans’ tendency to view categories as defined by some intrinsic, stable and essential property that defines category membership and is causally powerful in shaping other superficial features of the category members^21^. However, the role of such ‘essences’ may differ along ontological fields. Essentialism has been most intensively investigated in relation to natural kinds. Such beliefs about animals, for example, will lead us to assume that if something is born a dog, it will grow up to be a dog even if raised among cats, and will share most of its features with conspecifics. Moreover, adults will readily claim that an Irish setter is still an Irish setter even if its characteristically long hair is cut short. Importantly, such essentialist beliefs are present in humans from an early age (around the age 4), guiding conceptual development^22^.

There is wide scientific agreement that humans also apply such essentialist beliefs to social categories from early childhood. Specifically, a number of studies show that already pre-school aged children believe racial and ethnic categories to be absolute and determined by birth^23–26^. While beliefs about the biological determinism of category boundaries may seem intuitively appropriate for categories that are in fact related to genetic variation, there is another feature of essentialist reasoning that leads to significant bias: assuming a strong causal power of such distinctions in shaping other traits of the individual. Children (as well as adults) not only expect such categories to be innate but also expect members to share even non-kind relevant traits with in-groups rather than out-groups [e.g.^26,27^.

The pervasive nature of social essentialism is manifested in findings that children apply this belief-system even to kinds that lack any obvious biological ground. For example, children assume that the language someone speaks is determined by the native language of the birth parents rather than that of adoptive parents^28^, and that linguistic group membership is stable across development^29^. The results showing an early-emerging tendency to essentialize social categories and that these beliefs exhibit remarkable similarities with reasoning about animal kinds have led researchers to propose that social kinds are mistaken by the brain as appropriate input for a *folk-biology module*^24,30^. However, research findings that show significant cultural variation in which categories become essentialized contradict this notion^31^.

While essentialist beliefs undeniably yield incorrect stereotypes and predictions about individuals on several occasions, they also manifest a fundamental ambition of the human mind: to map stable correspondences between category membership and individual features in order to aid information processing and learning about the environment. Naturally, category-based inferences are most successful, if category boundaries are stable and mark causally powerful features. However, social categories represent a challenge in this respect. Although there are some salient category distinctions that are based on genetic variation (such as the Big 3, age, race and sex e.g.^2^), arguably, the real benefit of social categorization is to understand the more fine-grained organization of societies^7^. We can represent categories defined by nationality, religion, study groups, etc. Note that social kinds are not simply organized in a taxonomical structure but these categories overlap with each other and category boundaries may also be fleeting in time^5^. This presents a paradox for social categorization: while categorization in general is most effective if it can capture something stable about the given kind, one of the most important features of the social environment is that social groupings may change dynamically and thus, the relevance of distinction may also change rapidly.

A number of accounts have highlighted a function of social categorization that inherently invites this kind of dynamicity. Namely, that social category representations are formed first and foremost to map cultural (rather than biological) differences by keeping track of differences in knowledge states between individuals. Thus, people who seem to share knowledge with one-self in relevant domains will be categorized as in-group while those who appear ignorant will form the out-group^32–35^. While these theories bear significant resemblance to one another, some authors claim that certain aspects of shared knowledge, such as language, are prioritized over others^9^, while others emphasize that a more general sensitivity to shared knowledge helps to keep track of category boundaries in a dynamic way by allowing to flexibly switch between relevant differentiations^35^. This will help us to realize in an instant that co-workers should be viewed as in-group when discussing professional issues, but as out-group when we change to questions pertaining to our leisure activity. Arguably, social categorization processes fulfil their purpose if they can allow for this kind of flexibility.

In this paper, we set out to investigate whether children are sensitive to the above described important difference between social categories and other biological kinds: namely, that social categories are first and foremost social constructs and are, thus, not necessarily based on biologically determined stable characteristics. Our main goal was to examine children’s intuitive responses before they enter formal education. Since the effects of categorization in overt behaviour can be manifested robustly after the age of 3^14^ and our study may be considered exploratory in this particular question, we used a relatively wide age range (4 to 7 years).

We hypothesized that if such flexibility is indeed an inherent feature of social categorization, then children should be more likely to view transient markers as defining a social category than in the case of biological kinds (such as animals). Thus, we compared categorization of human and dog targets. The targets could vary along three dimensions: two permanent features (skin tone/breed and hair/fur colour) and one transient feature (shirt or harness colour). Skin was considered to be a relevant characteristic for human images as this distinction invites vast stereotypes in adults, while hair colour was added as a similarly salient feature that nonetheless differs from skin tone in at least two important ways: (1) there are considerably fewer stereotypes associated with hair colour; (2) in our participants’ social environment, hair colour varies between individuals, yet stays relatively stable over time for a particular person. The features of the dog targets were selected to match those of human images.

Moreover, we examined the same effects both in neurotypical children (Study 1) and children diagnosed with Autism Spectrum Disorder (Study 2). It is well-known that the social-cognitive abilities of individuals with ASD are significantly impaired, yet little is known specifically about how it affects social categorization. In general, research suggests that autism results in reduced abilities to extract abstract categorization rules from environmental stimuli, while categorization based on simple features may be intact^36–38^. Moreover, a handful of studies suggest that categorization of social stimuli may be specifically affected in ASD, whereby individuals fail to self-categorize at a higher level of abstraction and consequently do not develop a sense of social identity^39,40^. Although as of yet, empirical evidence about social categorization in ASD is scarce and indirect, we predicted that children with ASD may have more difficulty in representing social categories in terms of complex and flexible social relations and thus, may be more likely to focus on simple and stable characteristics.

## Materials and methods

### Ethics statement

This research was approved by the Research Ethics Committee of Eötvös Loránd University and was carried out in accordance with the Declaration of Helsinki. Informed consent was obtained from all parents of children in accordance with an Institutional Review Board-approved protocol.

### Participants

Study 1: 62 neurotypical children (33 girls, 29 boys; mean age ± SD: 65.0 ± 10.8 months, the ages ranged from 3.9 to 7.0 years) were recruited from state run preschools in Hungary.

Study 2: 18 children with a diagnosis of autism spectrum disorder (ASD group; 2 girls, 16 boys; mean age ± SD: 94.9 ± 35.8 months, the ages ranged from 3.7 to 12.8 years while mental age was between 3 and 8) and 17 neurotypical children (NT) matched in mental-age (mean age ± SD: 71.1 ± 15.9 months, the ages ranged from 3.7 to 8.0 years) and gender (2 girls, 16 boys) were recruited from a variety of schools, preschools and early intervention centers in Budapest (see Supplementary Table S1 for more details).

### Setup and visual stimuli

The stimuli were digitally drawn images of children and dogs presented on a computer screen (Study 1) or printed on laminated cards (Study 2). Four different sets of stimuli were used. Each set comprised of a group pair of either 2 groups of humans (Set A_H_; Set B_H_) or 2 groups of dogs (Set A_D_; Set B_D_). In each group pair, there was always one transient trait (shirt colour for humans or harness colour for dogs) that was consistent within groups but differed between groups. For instance, one group wore yellow shirts while the other wore green shirts. There were also 2 types of permanent traits for both humans and dogs that could be present in both groups or were divided between groups similarly to the transient trait. As an example, the group with yellow shirts all had lighter skin and the group with green shirts all had darker skin, while in both groups, half the humans had yellow hair and half had brown hair. As the example shows, the permanent traits were skin tone and hair colour for humans, while in dogs, these were fur colour and dog breed. The different kind of traits were presented in all combinations. The sets defined by these features are summarized in Supplementary Table S2.

### Procedure

The experimental procedures of Study 1 and Study 2 were different in some respects because the methods of Study 2 had to be adapted to suit nonverbal children as well as developmentally delayed children. The tests were carried out by a female experimenter. Children were seated at a table in front of a computer screen (Study 1) or the experimenter and the child sat on opposite sides of a table in a quiet room (Study 2). There was always a person known to the child present during the test, such as a teacher, therapist or other caretaker.

Note that children in Study 2 (ASD and NT groups) were told that they would be rewarded for their efforts. Namely, the following instructions were given (modified to fit the specific reward the child would receive) before the experiment started: “*You will receive stamps, and when the paper is full, we will be all done. What kind of stamp would you like, a bear or a mouse? When one of these rows is full* (*pointing to the paper*), *you will get to choose a sticker, that you can stick here.* (If personalized rewards were used, they would get that at these points as well.) *When we’re done with the game, you can keep this paper with all the stamps and stickers.*”

**Figure 1:**
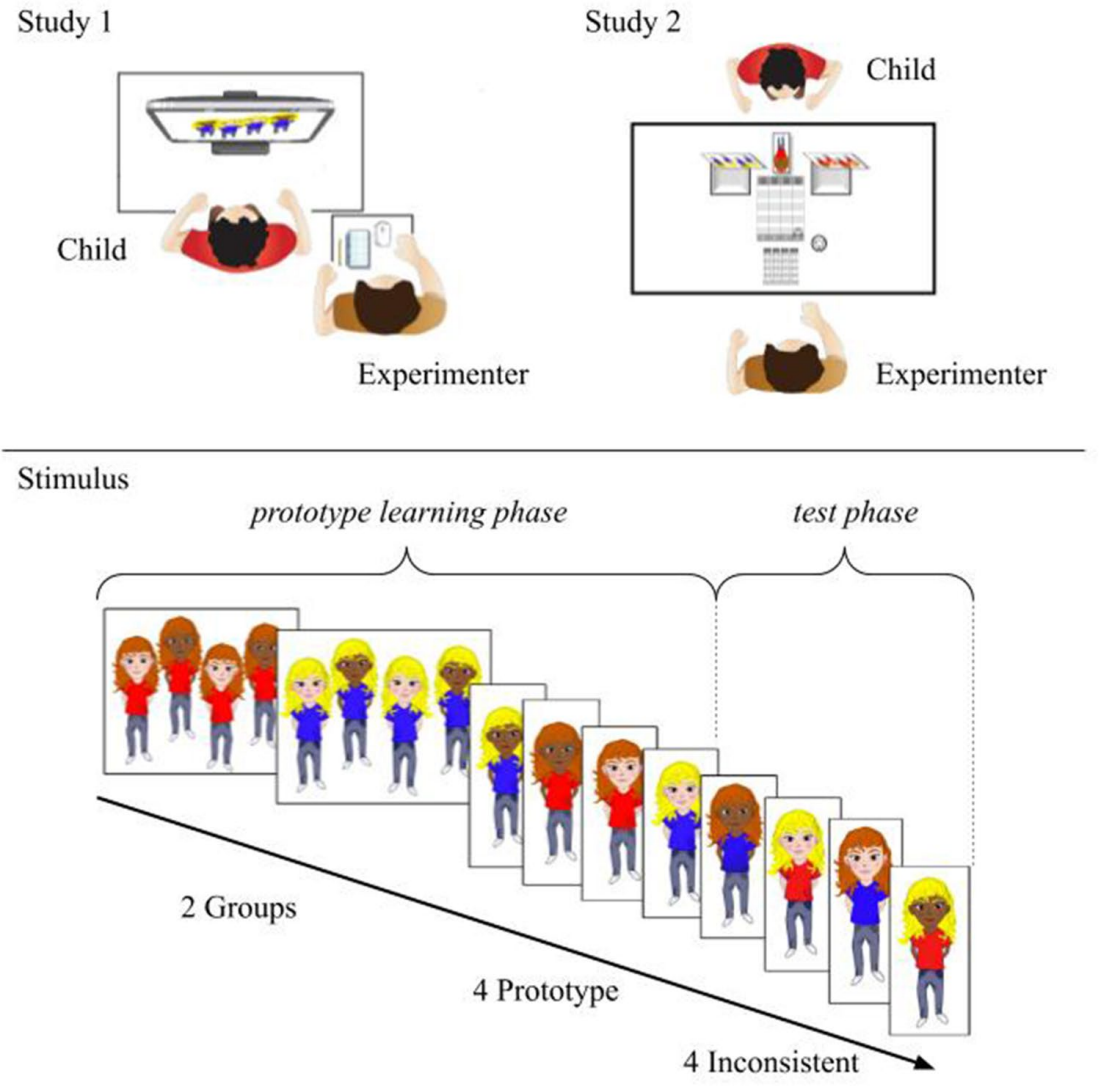
Experimental setup and example of stimuli (Human condition; Set AH, “Lomár” and “Helót”) in the order of presentation. 2 groups, 4 prototype and 4 inconsistent pictures shown.

Children participated in four different types of trials—trials involving different sets of stimuli (i.e. SetA_H_/B_H_/A_D_/B_D_—see Supplementary Table S2). The order of trials was randomized across participants with the restriction that human and dog sets were presented alternately. Each trial consisted of a prototype learning phase and a test phase (Fig. 1).

#### Prototype learning phase

The two groups were first introduced while also mentioning an activity that members of each group liked to do (E.g. “*Look! These are the ‘Lomár’-s. They like to play cards. And look! These are the ‘Helót’-s. They like to go fishing.”* For all group-activity pairings, see Supplementary Table S2). After presenting the groups of a trial, the experimenter explained the task to the child with the following instructions: “*I will show you some pictures of kids/dogs. Each is either a ‘Lomár’ or a ‘Helót’* (for example*). All you have to do is tell me whether the one I’m showing you is a ‘Lomár’ or a ‘Helót’*.”

At this point, the groups were no longer visible on the screen (Study 1) or on the table (Study 2). First, the 4 prototypes were shown, that could only fit into one of the groups (as explained above). The four individual prototypes were presented one by one in a randomized order. If the child verbally misidentified at least one (on-screen testing—Study 1) or did not place the prototype-card in the appropriate group (Study 2) the presentation of the trial was repeated from the beginning, reintroducing the groups maximum three times. Note that group names were relatively difficult to recall because they were meaningless. If the fourth attempt was still unsuccessful, the inconsistent images were not shown for that set and the trial was deemed unsuccessful, however, if they completed the prototype learning phase of the other stimulus sets without problem, the data from those were still used.

#### Test phase

If the child successfully completed the prototype learning phase, thus showing that they understood the task and could remember the groups and their names, they were presented with the inconsistent images one by one. No indication was given to the child that anything was different in these images from the previously presented prototypes. The four items that were inconsistent matched one group by their transient trait and the other by their permanent trait. For instance, a human with a red shirt, blond hair and either light or dark skin tone could be categorized as “Lomár” by the transient trait, but as a “Helót” by the permanent trait. In this portion of the test, there were no ‘right’ or ‘wrong” answers.

Children had to respond with either of the group names presented for that particular set (Study 1) or they had to place the card into one of the predetermined boxes (Study 2). However, if children did not respond, the question was repeated (at approx. 5 s intervals a maximum of 3 times: “*Where does it belong?/Put it where you think it belongs!*”). If children still failed to name one of the categories or failed to place the card into one of the predetermined boxes, the trial was ended and was excluded from the analyses. If the child was uncooperative or was not paying sufficient attention or showed signs of distress, the task was also terminated.

The entire test took approx. 12 min when the child cooperated, and no breaks were needed. However, no strict time limit was set and as many breaks could be taken as needed between trials with no set time limit, giving the child the option to choose when they wished to continue.

### Coding and data analyses

We analysed children’s first responses (choices). During the prototype learning phase, the responses were coded either right or wrong. During the test phase, the answer was coded as “permanent” if the child clearly indicated the category that shared the permanent trait (hair colour, skin tone, fur colour or breed) with the given exemplar. If the selected category matched the harness colour or the t-shirt colour that of the exemplar, the response was coded as “transient”.

Statistical analyses were executed using SPSS (IBM SPSS 23) software package. We used generalized linear mixed models (GLMM) with binomial distribution (binary logistic regression) to explore the differences in category choices across conditions in both Study 1 and Study 2. The model included a random grouping factor (subject IDs) and four fixed explanatory variables (factors). Note, however, that fixed factors included in the GLMMs in Study 1 and Study 2 were somewhat different. The following factors were included in the analyses for Study 1: Condition (dog figure/human figure), Permanent Label Type (hair/fur vs skin/breed), Trial Order (from 1 up to maximum 4) and Sex (boy/girl). The analyses for Study 2 included the factors: Condition, Permanent Label Type, Trial Order and Group (ASD/Neurotypical). Moreover, age in months (Study 1) and mental age in years (Study 2) were included as covariates and the models also contained all two-way interactions.

Non-significant interactions and main effects were removed from the model in a stepwise manner (backward elimination technique). We also calculated parameter estimates (B) and standard errors (SE) for significant variables and interactions. In order to better understand the interaction effects, we conducted post-hoc tests. We tested for moderation effect with the PROCESS macro for SPSS^41^. Our moderation model included age in months as independent variable (focal predictor) and Choice (binary response variable) as dependent variable with Sex as the moderator. Furthermore, Condition, Permanent Label type and Trial order were included as statistical controls. For within group, within Permanent Label type and within condition comparisons, we used pairwise estimated marginal means contrast analysis with Bonferroni corrections. All tests were two-tailed and the α value was set at 0.05.

## Results and discussion

### Study 1: Social categorization of dogs and humans in neurotypical children

*Prototype learning phase:* All participants completed at least one trial; 50 children (80.64%) successfully completed all four trials, others, however, failed to complete 1–3 trials (three failed trials: 4 children, two failed trials: 1 child, one failed trials: 7 children). Children, who had at least one failed trial (N = 12, N = 4 boys and N = 8 girls) were younger (Mean age: 454.3 months) than those who completed all trials for their first attempt (Mean age: 68 months).Overall, they performed well in the prototype learning phase (82.1% of their responses were correct on the first attempt).

The GLMM analysis revealed a main effect of Condition (F_1,904_ = 6.132 *p* = 0.013, human figure vs dog figure condition, regression coefficient B: 1.823, 95% CI [0.381, 3.270], η^2^ = 0.449) and Age (F_1,904_ = 14.044, *p* < 0.001, B: − 0.009, 95% CI [− 0.025, 0.007], η^2^ < 0.01) and the interaction of these two factors was also significant (F_1,904_ = 6.349, *p* = 0.012, B: − 0.029, 95% CI [− 0.051, − 0.006], η^2^ < 0.01). These results show that older children were generally more successful in the prototype learning phase, but this effect was mainly driven by trials with dog stimuli; prototype categorization of human images remained largely the same across ages. All other main and interaction effects were non-significant (*p* > 0.05 for all).

**Figure 2:**
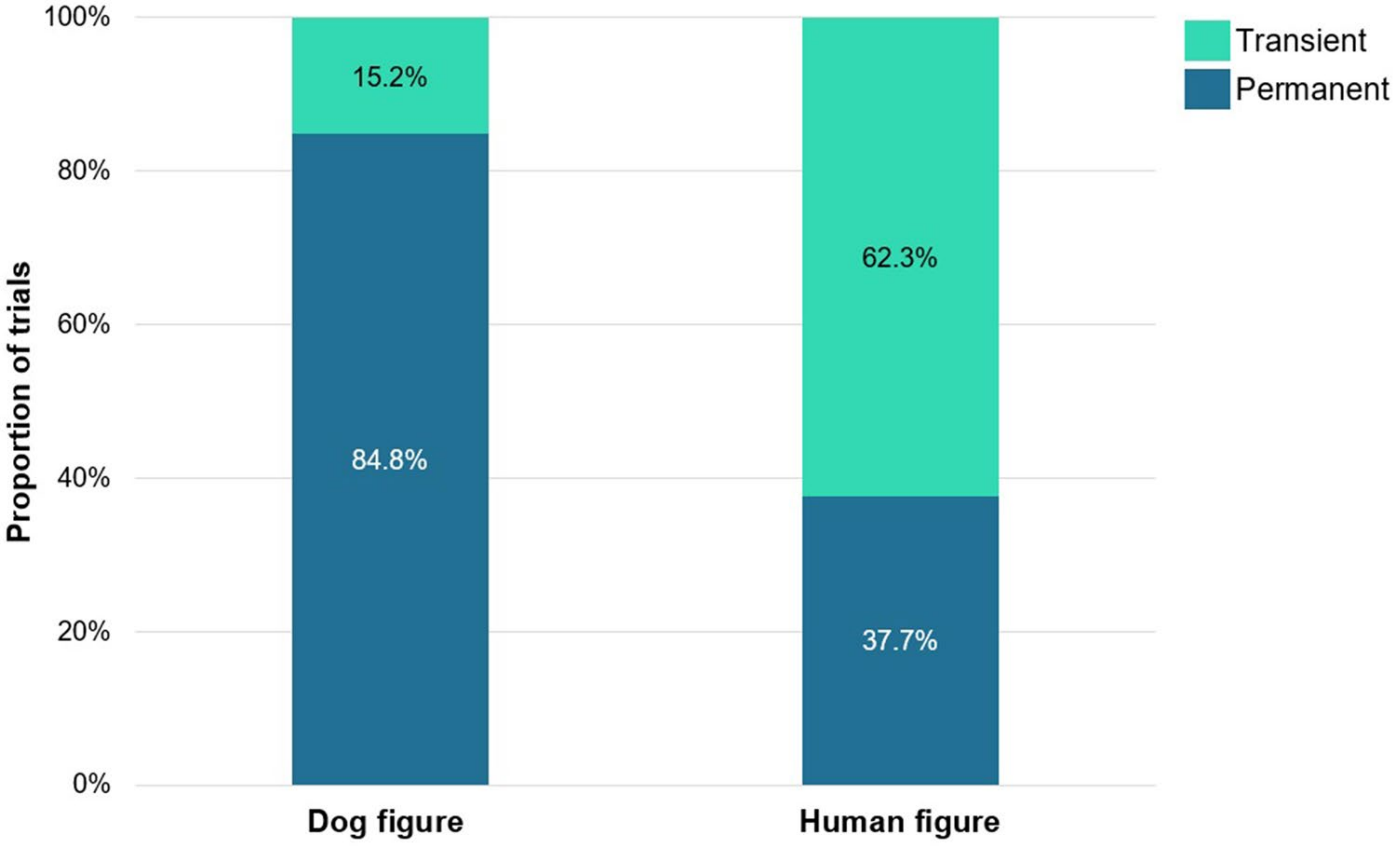
Categorization of human and dog images based on transient and permanent features. The graph depicts the percentage of trials where a specific type of choice was made.

*Test phase:* The GLMM analysis showed a significant main effect of *Condition* (dog vs. human figure; F_1,891_ = 170.259; *p* < 0.001, η^2^ = 0.241) on children’s responses suggesting that they tend to categorize human and dog images differently (human figure vs dog figure condition, B: − 2.045, 95% CI [0.081, 0.206]) (Fig. 2). More specifically, children showed a robust tendency to categorize dogs based on permanent features (fur colour, breed—in 84.8% of the total responses) whereas they showed an opposite response bias in the ‘human figure condition’ (preference for the transient trait in 62.3% of the total responses—Wilcoxon signed rank test, *p* = 0.004).

**Figure 3:**
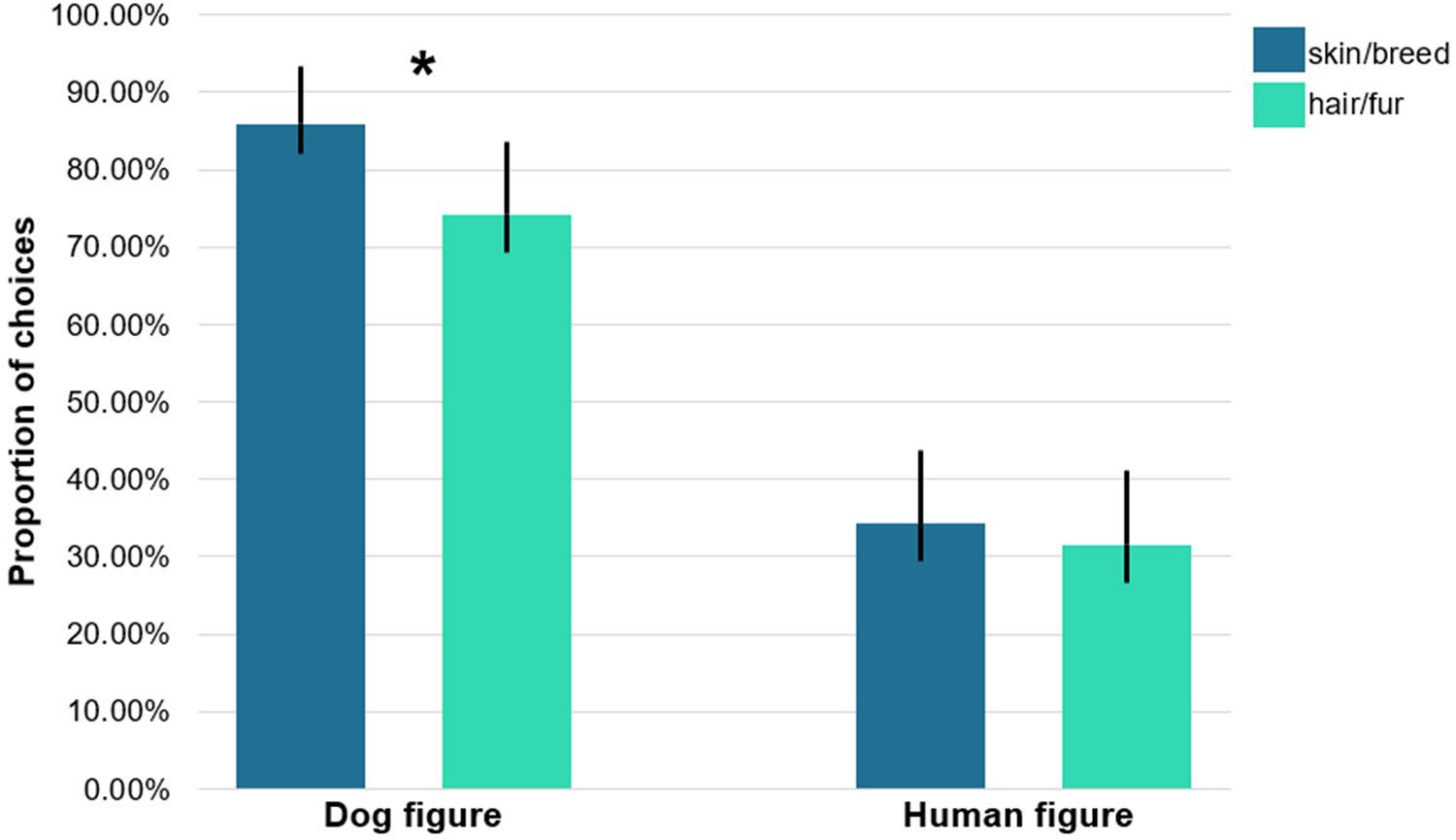
Mean proportion (+ CI) of trials where children categorized based on the permanent trait shown separately for the different kind of permanent markers. Asterisks represent significant differences between the use of Permanent Label types in the Dog figure condition. The use of skin/breed, as permanent label was significantly different between conditions (GLMM of Choice, effect of Permanent Label type: F_1,891_ = 10.018; *p* = 0.002), driven by a more prevalent use of breed label than fur in the Dog figure condition (hair/fur vs skin/breed, contrast estimate: − 0.116, t(891) =  − 3.475; *p* = 0.001, 95% CI [− 0.182, 0.050]), but not in the Human figure condition (hair/fur vs skin/breed, contrast estimate: − 0.032, t(891) =  − 0.607; *p* = 0.544, 95% CI [− 0.135, 0.072]). Asterisks represent significant differences based on post hoc analysis.

*Permanent Label Type* also had a significant main effect on children’s responses (hair/fur vs skin/breed; F_1,891_ = 10.018; *p* = 0.002, B: 0.495, 95% CI [0.693, 3.888], η^2^ = 0.018) and there was a significant interaction between *Condition* and *Permanent Label Type* (F_1,891_ = 6.104; *p* = 0.014, B: − 0.914, 95% CI [0.194, 0.829], η^2^ = 0.059). Children were more likely to categorize dogs based on the permanent trait if that was indicated by breed than by fur colour, but this tendency was not observed when the respondent categorized human figures (Fig. 3).

**Figure 4:**
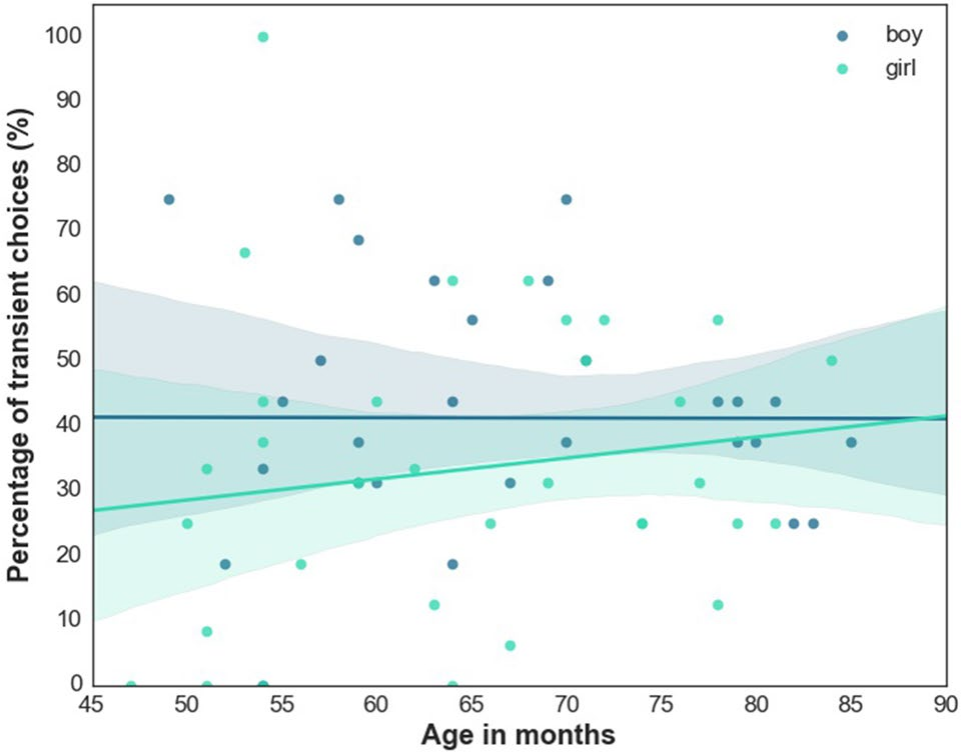
Age and gender interactive effects on children’s tendency to rely on transient marker when categorizing humans and dogs. Individual dots represent the proportion of ‘transient’ category choices for individual participants.

Interestingly, *Sex* also had a significant main effect (F_1,891_ = 5.886; *p* = 0.015, girl vs. boy B: 4.426, 95% CI [2.329, 3002.350], η^2^ = 0.598) on children’s category choices. Girls were less likely to rely on transient markers compared to boys, suggesting that their choices were driven by skin tone or hair colour when categorizing humans, or breed and fur colour in the case of trials involving dogs. Note, however, that there was a significant *Sex* x *Age* interaction effect (F_1,891_ = 4.409; *p* = 0.036, B: − 0.057, 95% CI [0.895, 0.996], η^2^ < 0.01), suggesting that these gender differences became less pronounced with age (Fig. 4).

In order to reveal the interaction effect, we tested for the moderation effect of Sex including Age in months as independent variable (focal predictor) and Choice (binary response variable) as dependent variable in the model (the Condition, Permanent Label type and Trial order were included as statistical controls). According to the results, the overall moderation model had a significant effect (χ^2^_(6)_ = 248.67, *p* < 0.001). The overall Age in months x Sex interaction effect was also significant: b = − 0.037, χ^2^_(1)_ = 6.053, *p* = 0.014 CI = − 0.0679 ÷ 0.0075. We further used the Johnson-Neyman technique^42^ to probe for interaction effects and to identify ranges of age for which the interaction effect is significant. Hayes’ PROCESS macro^41^ incorporates the Johnson-Neyman technique^43^. One such region of significant moderation, from 47.00 to 71.86 months values of Age was identified. The effect of Sex on Choice is significantly at or below 71.86, but nonsignificant above that (see Figure S1).

The GLMM analysis showed a main effect of *Trial Order* (F_3,894_ = 3.607 *p* = 0.013, 4 vs. 1 B ± SE: 2.996, 95% CI [0.630, 634.346], η^2^ = 0.405, 4 vs 2 B: 2.011, 95% CI [0.232, 240.243], η^2^ = 0.235, 4 vs 3 B: − 2.862, 95% CI [0.002, 1.926], η^2^ = 0.384) and the *Trial Order* x *Age* (F_3,891_ = 4.953 *p* = 0.002) as well as the *Trial Order *× *Permanent Label Type* (F_3,891_ = 3.200; *p* = 0.023, skin/breed * Trial order = 1: η^2^ = 0.179, skin/breed * Trial order = 2, η^2^ = 0.018, skin/breed * Trial order = 3, η^2^ = 0.008) interactions were also significant. The pattern of responses indicate that children were slightly more likely to categorize based on transient features in later trials. Moreover, in the first trial, children were more likely to categorize based on the permanent marker if it was indicated by skin tone or breed (as opposed to hair or fur colour) but this difference became less pronounced as the trials progressed. The main effect of *Age* and other interaction effects were not significant (*p* > 0.05 for all). We have provided additional information, including the non-significant main effects and interaction effects for both studies, along with the parameter estimates in the supplementary material (see Table S3–S6 for the results of the GLMM and Table S7–S9 for parameter estimates).

These results clearly indicate that typically developing 4 to 7-year-old children assign different significance to permanent and transient features when categorizing humans and animals (in this case, dogs). Specifically, children have a robust tendency to categorize dogs based on permanent features, such as fur colour or breed. In contrast, children were much more likely to view the transient trait as the basis on which humans should be categorized, choosing this trait on more than 60% of the inconsistent images during the test phase. The presence of such different tendencies was supported by the statistical analyses with a fairly large effect size. While our main hypothesis was confirmed, a number of other effects have also emerged. Overall, when groups could be differentiated by skin tone or breed, participants were more likely to categorize based on permanent features (although effect sizes for these analyses were relatively small). However, while the skin tone of human figures had only a small (if any) effect, breed seemed to make more of a difference in categorizing dogs. We also found that girls were more likely to focus on permanent features, a trend that diminished with age. Importantly, sex was not found to be in a significant interaction with condition, showing that both girls and boys used differential strategies in categorizing humans and animals. These results suggest that neurotypical children show an understanding of the socially constructed nature of social categories and do not conflate them with biological categories.

### Study 2: Social categorization in children with ASD

#### Prototype learning phase

Only 4 children from the ASD group had difficulties in one trial with categorizing the prototypes on their first attempt and there was an additional ASD participant who had difficulties in three out of four trials. Note, however, that all children with ASD completed each trial successfully and they performed very well in the prototype learning phase (95.6% of their responses were correct on the first attempt). There were only two children from the neurotypical group who made incorrect responses (99.3% of the responses were correct on the first attempt). The GLMM analyses of the prototype learning performance showed no main or interaction effects of *Condition, Permanent Label Type, Group, Mental Age* or *Trial Order* (*p* > 0.05 for all). The results indicate high category learning performance both in the experimental (ASD) and the control (NT) group.

#### Test phase

**Figure 5:**
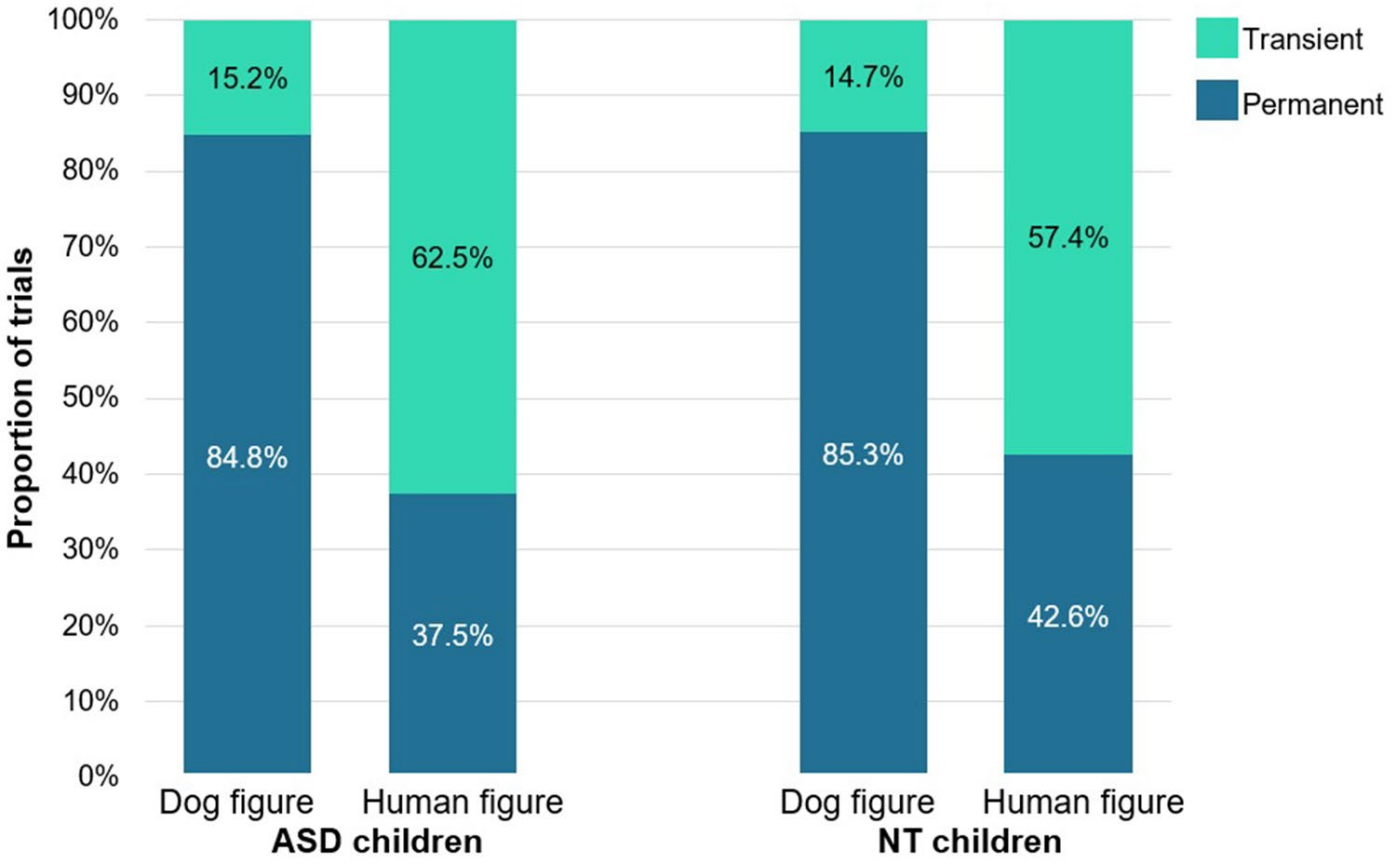
Categorization of human and dog images based on transient and permanent features. The graph depicts the percentage of trials where a specific type of choice was made by children with ASD and neurotypical (NT) participants.

The GLMM analysis showed that *Condition* had a significant main effect on children’s category choices (human vs. dog figure; F_1,516_ = 92.084; *p* < 0.001, B: − 2.247, 95% CI [0.052, 0.215], η^2^ = 0.276). Both neurotypical and autistic children were more likely to rely on permanent traits (breed/fur) when categorizing dogs, while transient traits proved to be more important for humans (Fig. 5).

**Figure 6:**
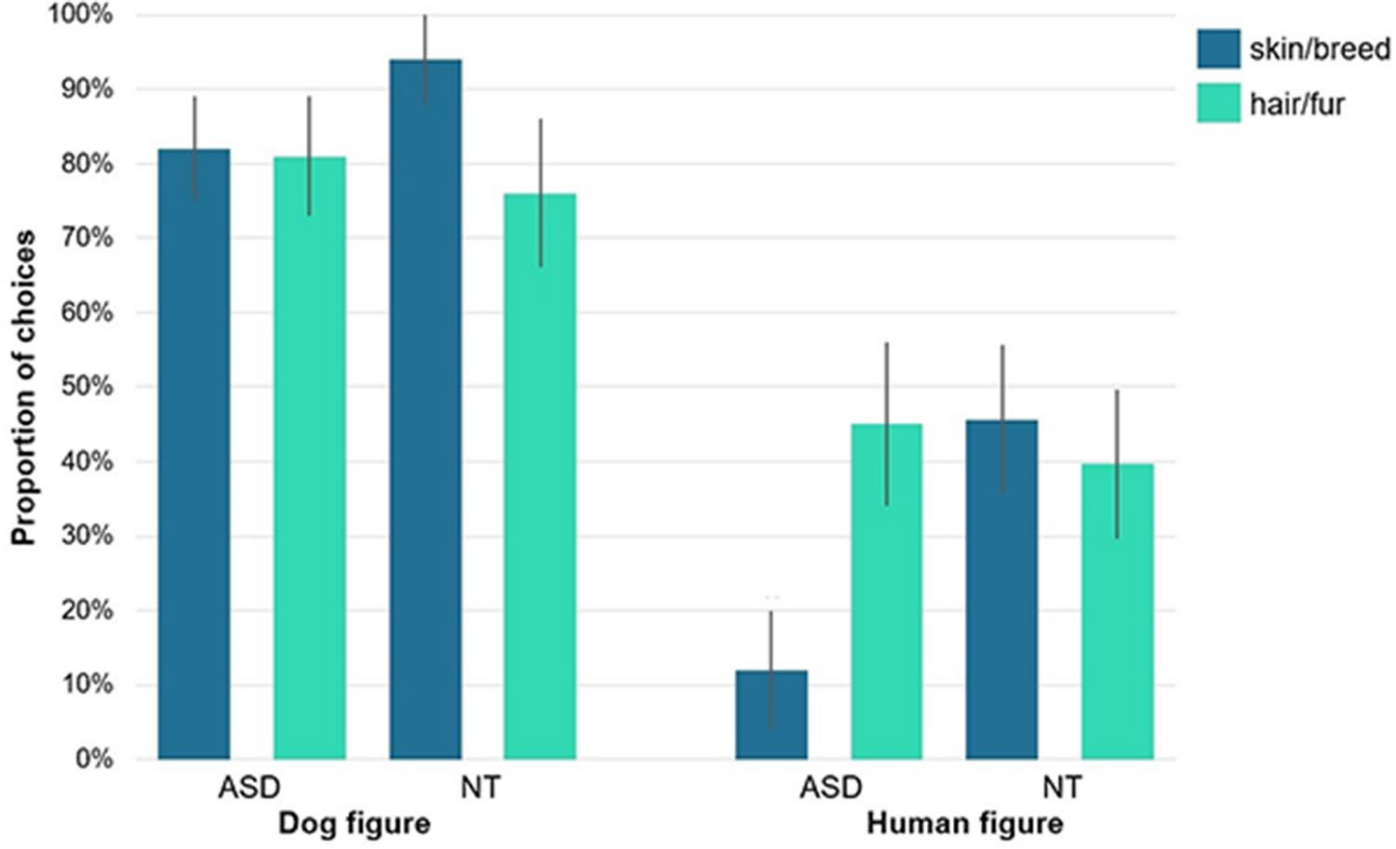
Mean proportion (+ CI) of trials where neurotypical (NT) and autistic (ASD) children categorized based on the permanent trait shown separately for the different kind of permanent markers. Pairwise comparisons using estimated means-contrasts show that children in the NT group were more likely to categorize based on permanent traits using skin/breed as Permanent label type (skin/breed vs hair/fur contrast estimate: − 0.349, t(516) =  − 3.446; *p* = 0.001, 95% CI [− 0.549, − 0.150]), while the opposite pattern was observed in the ASD group (skin/breed vs hair/fur contrast estimate: 0.232, t(516) = 2.519, *p* = 0.012, 95% CI [0.051, 0.413]). Post-hoc comparisons using estimated means-contrasts show that in the dog figure condition children of both groups were more likely to categorize based on permanent traits using skin/breed as Permanent label type (skin/breed vs hair/fur contrast estimate: − 0.109, t(516) =  − 2.378; *p* = 0.018, 95% CI [− 0.198, − 0.018]), while the opposite, although non-significant results were observed in the human figure condition (skin/breed vs hair/fur contrast estimate: 0.079, t(516) = 1.010, *p* = 0.313, 95% CI [− 0.074, 0.232]).

Although the main effects of Group and Permanent Label Type were not significant (*p* > 0.05) there were significant interactions between Permanent Label Type and Condition (F_1,516_ = 8.629; *p* = 0.003, B: − 1.769, 95% CI [0.052, 0.557], η^2^ = 0.191), and between Permanent Label Type and Group (F_1,516_ = 20.344; *p* < 0.001, B: − 3.062, 95% CI [0.012, 0.178], η^2^ = 0.415). It seems that neurotypical children were more likely to categorize dogs based on the permanent trait when it was indicated by breed, while children with autism assigned more weight to hair colour than skin tone (Fig. 6).

The main effect of *Mental Age* was also not significant at the *p* < 0.05 level, but we found a significant interaction between *Mental Age* and *Permanent Label Type* (F_1,516_ = 5.827; *p* = 0.016, B: − 0.437, 95% CI [0.453, 0.922], η^2^ = 0.014). This effect is mainly driven by older children’s tendency to overwhelmingly choose the permanent marker as the basis for categorization when skin tone/breed indicates group membership. We also found a main effect of *Trial Order* (F_3,512_ = 3.655 *p* = 0.012, 4 vs 1 B: 0.030, 95% CI [0.451, 2.089], η^2^ < 0.01, 4 vs 2 B: 0.080, 95% CI [0.444, 2.644], η^2^ < 0.01, 4 vs 3 B: 1.143, 95% CI [1.345, 7.312], η^2^ = 0.090), showing that children chose the transient trait more often in trial 3 than in the other three trials. Other main effects and interaction effects were not significant (*p* > 0.05 for all).

## General discussion

The present studies were designed to test whether children are sensitive to the fact that social categories are first and foremost social constructs that are characterized with flexible category boundaries. To investigate this question, we tested whether children would be more likely to categorize individuals based on transient, rather than permanent features. We hypothesized that an opposite pattern would be observable for animal categories, since relevant groupings of animals usually correspond to biological distinctions. Our results support these hypotheses. We have found that the dominant categorization strategy for dog stimuli was to match the individual to the group based on permanent features, such as breed or fur colour. Although less robustly, an opposite pattern of results emerged in the case of human stimuli: on the majority of occasions, children assumed that an individual belongs with those that share the transient trait (t-shirt) with them.

Overall, the same pattern of results was observed for children with ASD and neurotypical children in Study 2: participants in both groups were more likely to categorize images of humans based on the transient trait, while they overwhelmingly chose the permanent trait as the basis for categorization when categorizing dogs. However, there were also some minor differences in the strategies that we observed. Children in the two groups showed a difference in terms of which permanent trait they put more emphasis on. While neurotypical children were more likely to categorize based on skin tone or breed than hair or fur colour, the opposite pattern was observed in children with autism. Thus, the observed effect does not reflect any differences that are specific to social categorization. One possibility is that this difference emerges due to a perceptual bias that has been suggested to characterize autism spectrum disorder^44–46^: children with ASD may be more likely to be categorizing based on features that do not require the integration of visual information received from different parts of the scene. For example, while skin tone can be best determined by considering all parts of the body that are not covered with clothes, hair colour creates an arguably more salient distinction on the pictures locally. Moreover, while neurotypical children may be most likely focus on the face area of a picture depicting a human, this is not necessarily the case for children with ASD, making skin tone more salient to the former group. This difference in the need for feature integration is even more pronounced in the case of dogs: breed is a category distinction that cannot be grasped by one specific feature but to determine it, holistic visual processing is required. Nonetheless, it is possible that this modulation effect of permanent trait type results from other perceptual (e.g. saliency effects to which children with ASD may be more susceptible) or conceptual biases. Future studies could clarify this question.

Although age did modulate the pattern of results to some degree in interaction with other variables, we did not find very robust age differences (a tendency to favour transient features with age was only observed in the case of girls, but not boys and only for a certain age range, between 41 and 71 months and effect sizes were also relatively small). This is interesting in light of the fact that the age range covered in our study was relatively wide and studies usually indicate that racial categorization becomes more robust during this period e.g.^23^. However, note that the sample size does not warrant any strong conclusions about the lack of developmental effects, therefore, it remains an open question whether children would switch strategies in social categorization with age.

It should also be noted that in our study, only one transient feature was used for human images: shirt colour. Thus, we cannot be absolutely certain whether this feature specifically carries some perceptual characteristics that lend it an ideal marker for categorization or it is seen more generally as one of the many possible ways that socially constructed categories can be indicated. Shirt colour seemed an ideal candidate for the transient feature as it is similarly salient as hair colour or skin tone, yet carries a symbolic meaning: children from an early age are familiarized with the idea that group cohesion is often signalled by similar clothing as in the case of sports teams, for example. Thus, there is a possibility that the effect observed here cannot be generalized to any transient feature but only to those with which children have already gained experience. However, it is also important to note that these associations that children may have formed between clothing and social groups are not specific perceptual correlations: children probably do not have any stable representations that would connect blue shirts with any particular social groups and they probably have ample experience with people wearing blue shirts without it being a meaningful signal of group affiliations. Rather, they have gained an understanding that symbolic cues that are at the same time transient (as clothes are changed all the time) have a stronger relevance in social categorization than other similarly salient and stable perceptual features. Because of this, we would argue that even if the effect was not equally strong with just any transient marker, the present results still cannot be accounted for by simple statistical learning specific to shirt colour.

On a similar note, it is also important to consider that it is difficult to find traits that unambiguously signal biologically set stable properties that nonetheless lack any cultural significance. Cultural learning will inevitably result in associating new meaning with categories defined by skin tone or hair colour (consider the not very sophisticated, yet common jokes about blonde-haired women). Thus, these traits may gain symbolic significance with development.

Note that we also found that trial order modulated the results, even though we did not predict any specific learning effects. Although this is speculative, we believe that the most likely explanation for this is that when children are faced with a similar task repeatedly, they will try different strategies in order to find the „correct” solution. Since the experimenter did not give any direct feedback on their responses (in order to avoid biasing them), they may have inferred that they should try other strategies as well. Importantly, trial order was randomized in our experiment, therefore it cannot systematically bias our results.

In general, across the two studies, we found that our main hypotheses were supported by relatively large effect sizes in the statistical analyses (e.g. the effect of condition in both experiments, the interaction of Permanent Label type with other variables – although as a main effect, it produced only weak effects). However, the effect of certain control variables also turned out to be quite large (e.g. Sex, Trial order in Study 1). Nonetheless, caution should be taken in interpreting the effect sizes of the study since sample sizes were relatively low (similarly to other studies in the field e.g.^36,37^) and consequently, the analyses may not produce perfectly reliable estimates of effect sizes^47^.

The result that social categories are viewed as social constructs from an early age is in line with studies that highlight the role of social category representations in guiding cultural learning. It has been repeatedly shown that children from the age of 14 months prefer to acquire culturally relevant knowledge from linguistic in-group members^13–16,48^, but do not necessarily use these distinctions to make inferences about the idiosyncratic preferences of category members^13^. Moreover, Krieger and colleagues^49^ failed to find similar selectivity in learning when potential teachers differed along race rather than language use. These findings corroborate our result that children from an early age intuitively understand the role of social categories in maintaining the coherence of cultures and that not all of the observable differences among individuals carry the same weight in this respect.

These results, however, do not necessarily indicate that social categories cannot be essentialized. There is compelling evidence that the process through which children come to essentialize certain categories is highly susceptible to social input^50,51^. In these studies, the use of generic language while introducing novel social categories led both children and adults to form essentialist beliefs about the category. Nonetheless, it is possible that not every dimension of essentialist beliefs will be applied to each of the socially constructed categories: children need not think that shirt colour reflects innate differences among individuals, yet they may view the distinction as inductively powerful. This idea is in line with the general finding in the literature that there is considerable variance in which aspect of essentialism children apply to social categories^52^.

In closing, it should be mentioned that while social categorization is generally used as an umbrella term for every distinction created between humans, it is possible that multiple representational systems stand behind these processes. Representations of some primary, biologically relevant categories, such as gender or age, may be created through different (evolutionary more ancient) mechanisms than those about sports teams. Nonetheless, we argue that to navigate through the complex structure of human societies, humans have to be equipped with a cognitive faculty that is prepared to handle the mesh network of often overlapping social groups. Humans should not only be able to extract generic knowledge about fellow humans from category information, but they should also be ready to switch to an alternative way of categorizing people in an instant if contextual cues warrant it. Our study, despite the aforementioned limitations associated with small sample size, shows that this flexibility in categorization may already be present in childhood and thus appears worthy of further investigation.

## Supplementary Information

Supplementary Information

## Data Availability

The data files for the presented studies can be accessed under the following link: https://osf.io/y8gqz/.
