# Supplementary material for: Social categorization based on permanent versus transient visual traits in neurotypical children and children with autism spectrum disorder
Source: Sci Rep. 2021 Mar 22;11:6549. doi: 10.1038/s41598-021-85924-w (PMC7985514; doi:10.1038/s41598-021-85924-w)

**Supplementary material**

**Social categorization based on permanent vs transient visual traits in neurotypical children and children with autism spectrum disorder**

Orsolya Kiss^1,4^**^†^**, Katalin Oláh^2^**^†^**^*^, Lili Júlia Fehér^3^, József Topál^1^

^1^ Institute of Cognitive Neuroscience and Psychology, Research Centre for Natural Sciences, 2 Magyar Tudósok krt, Budapest, Hungary

Budapest, Hungary

^2^MTA-ELTE Social Minds Research Group, Eötvös Loránd University, 46 Izabella u., Budapest, Hungary,

^3^ Pázmány Péter Catholic University, 1 Mikszáth Kálmán tér, Budapest, Hungary

^4^ Department of Cognitive Science, Budapest University of Technology and Economics, 1 Egry József u., Budapest, Hungary

†These authors have contributed equally to this work and should be considered co-first authors.

* corresponding author: Katalin Oláh, [olah.katalin@ppk.elte.hu](mailto:olah.katalin@ppk.elte.hu)

**Supplementary material**

**Table S1.** Matching of the Neuropypical control group to the ASD participants on mental age. In most cases, mental age was not included in the diagnostic forms of the children, so their special education teachers were asked to give an approximation about the child’s mental age (in years). The control group was thus matched based on age in years.

| **children with ASD** | | | **neurotypical children** | | |
| --- | --- | --- | --- | --- | --- |
| chronological age (years) | gender | estimated mental age | chronological age (years) | gender | estimated mental age |
| 3.7 | boy | 3+ | 3.7 | boy | 3+ |
| 5.9 | boy | 3+ | 3.8 | boy | 3+ |
| 4.2 | boy | 4+ | 4.5 | boy | 4+ |
| 4.7 | boy | 4+ | 4.7 | boy | 4+ |
| 7.1 | boy | 4+ | 4.8 | boy | 4+ |
| 4.8 | boy | 5+ | 5.1 | boy | 5+ |
| 5.6 | boy | 5+ | 5.7 | boy | 5+ |
| 7.4 | boy | 5+ | 5.8 | boy | 5+ |
| 6.6 | boy | 6+ | 6.0 | boy | 6+ |
| 7.0 | boy | 6+ | 6.5 | boy | 6+ |
| 9.8 | boy | 6+ | 6.8 | boy | 6+ |
| 11.0 | girl | 6+ | 6.8 | girl | 6+ |
| 10.8 | girl | 7+ | 7.0 | girl | 7+ |
| 11.3 | boy | 7+ | 7.0 | boy | 7+ |
| 11.4 | boy | 7+ | 7.2 | boy | 7+ |
| 12.8 | boy | 7+ | 7.6 | boy | 7+ |
| 10.8 | boy | 8+ | 8.0 | boy | 8+ |
| 5.2 | boy* | 4+ | no matched pair | | |

* This child with an ASD diagnosis was excluded from the analysis as he showed signs of distress when entering the testing area.

**Table S2.** Stimulus sets used in Study 1 & Study 2

| **Condition** | **Stimulus Set** | **Group Name** | **Group decription** | **Permanent trait (consistent)** | **Transient trait (consistent)** | **Permanent trait (variable)** |
| --- | --- | --- | --- | --- | --- | --- |
| Human | Set A_H_ | "Lomár" | *“They like to play cards”* | Brown hair | Red T-shirt | Skin tone |
|  |  | "Helót" | *“They like to go fishing”* | Blond hair | Blue T-shirt | Skin tone |
|  | Set B_H_ | "Pupák" | *“They like to go hiking”* | Light skin tone | Yellow T-shirt | Hair color |
|  |  | "Luppancs" | *“They like to go swimming”* | Dark skin tone | Green T-shirt | Hair color |
| Dog | Set A_D_ | "Cserbengóc" | *“They like to play fetch”* | Small sized, short haired (Dachshund) | Red harness | Fur color |
|  |  | "Rekkencs" | *“They like to chase each other”* | Medium sized, long haired (Border collie) | Blue harness | Fur color |
|  | Set B_D_ | "Bendőc" | *“They like to go hunting”* | Grey fur | Yellow harness | Breed |
|  |  | "Rikács" | *“They like to bark”* | Brown fur | Green harness | Breed |

**Summary of GLMM analyses (Table S3-S6)**

**Table S3. Study 1 – Prototype learning phase**

The *Performance* (binary) variable was analysed by generalized linear mixed-effect models (GLMMs) with binomial distribution. To control for repeated measures, we applied random intercept GLMM with subject ID included as a random grouping factor. The fixed explanatory variables included Condition (factor with two levels), Permanent Lable type (factor with two levels), Trial Order (factor with four levels), Sex (factor with two levels), and age in months as a covariant. The model included all two way interactions of the variables. All tests were two-tailed and the α value was set at 0.05. Nonsignificant variables were removed sequentially, in a backward stepwise procedure after the removal of the least significant. The table shows all the main and interaction effects along with their significance in the model (in case of nonsignificant effects it shoes the p value before the elimination). Asterisks represent significant values (∗ p < 0.05, ∗∗ p < 0.01)

| **GLMM** | **Variable** | **F** | **df1** | **df2** | **p** |
| --- | --- | --- | --- | --- | --- |
| Main effects | Condition | 6.132 | 1 | 904 | 0.013* |
|  | Permanent Label type | 0.000004 | 1 | 896 | 0.998 |
|  | Sex | 2.997 | 1 | 897 | 0.084 |
|  | Trial order | 1.615 | 3 | 901 | 0.184 |
|  | age in months | 14.044 | 1 | 904 | <0.01** |
| Interaction effects | Condition x Permanent Label type | 1.342 | 1 | 892 | 0.247 |
|  | Condition x Trial order | 0.782 | 3 | 888 | 0.504 |
|  | Condition x age in months | 6.349 | 1 | 904 | 0.012* |
|  | Condition x Sex | 0.031 | 1 | 882 | 0.861 |
|  | Permanent Label type x Trial order | 1.621 | 3 | 893 | 0.183 |
|  | Permanent Label type x age in months | 0.208 | 1 | 887 | 0.649 |
|  | Permanent Label type x Sex | 0.173 | 1 | 886 | 0.677 |
|  | Trial order x age in months | 2.239 | 3 | 898 | 0.082 |
|  | Trial order x Sex | 0.506 | 3 | 883 | 0.678 |
|  | Sex x age in months | 0.571 | 1 | 891 | 0.450 |

**Table S4. Study 1 – Test phase**

The *Choice* (binary) variable was predicted using generalized linear mixed models (GLMM) fitted with a binomial distribution with random intercept (using the subject’s ID). The model included the same explanatory variables and interaction effects as in the Prototype learning phase. Asterisks represent significant values (∗ p < 0.05, ∗∗ p < 0.01)

| **GLMM** | **Variable** | **F** | **df1** | **df2** | **p** |
| --- | --- | --- | --- | --- | --- |
| Main effects | Condition | 170.259 | 1 | 891 | < 0.001** |
|  | Permanent Label type | 10.018 | 1 | 891 | 0.002** |
|  | Sex | 5.886 | 1 | 891 | 0.015* |
|  | Trial order | 3.607 | 1 | 891 | 0.013* |
|  | age in months | 0.003 | 1 | 891 | 0.958 |
| Interaction effects | Condition x Permanent Label type | 6.104 | 1 | 891 | 0.014* |
|  | Condition x Trial order | 2.326 | 3 | 888 | 0.073 |
|  | Condition x age in months | 1.534 | 1 | 886 | 0.216 |
|  | Condition x Sex | 1.106 | 1 | 885 | 0.293 |
|  | Permanent Label type x Trial order | 3.200 | 3 | 891 | 0.023* |
|  | Permanent Label type x age in months | 0.032 | 1 | 881 | 0.857 |
|  | Permanent Label type x Sex | 2.179 | 1 | 887 | 0.140 |
|  | Trial order x age in months | 4.953 | 3 | 891 | 0.002** |
|  | Trial order x Sex | 1.000 | 3 | 882 | 0.392 |
|  | Sex x age in months | 4.409 | 1 | 891 | 0.036* |

**Table S5. Study 2 – Prototype learning phase**

The *Performance* (binary) variable was analysed by generalized linear mixed-effect models (GLMMs) with binomial distribution. Incuded factors: Condition (factor with two levels), Permanent Label type (factor with two levels), Group (factor with two levels), Trial order (factor with four levels). The model included the mental age as covariant, and the subject’s ID as random intercept. The table shows all the main and interaction effects along with their significance in the model (in case of nonsignificant effects it shows the p value before the elimination). Asterisks represent significant values (∗ p < 0.05, ∗∗ p < 0.01)

| **GLMM** | **Variable** | **F** | **df1** | **df2** | **p** |
| --- | --- | --- | --- | --- | --- |
| Main effects | Condition | 0.344 | 1 | 539 | 0.568 |
|  | Permanent Label type | 1.330 | 1 | 540 | 0.249 |
|  | Group | 2.524 | 1 | 541 | 0.113 |
|  | Trial order | 1.080 | 3 | 536 | 0.357 |
|  | mental age | 2.735 | 1 | 542 | 0.099 |
| Interaction effects | Condition x Permanent Label type | 0.0001 | 1 | 524 | 0.990 |
|  | Condition x Trial order | 0.001 | 3 | 518 | 1.000 |
|  | Condition x mental age | 0.345 | 1 | 532 | 0.557 |
|  | Condition x Group | 0.00007 | 1 | 525 | 0.983 |
|  | Permanent Label type x Trial order | 0.029 | 3 | 526 | 0.993 |
|  | Permanent Label type x mental age | 0.371 | 1 | 535 | 0.543 |
|  | Permanent Label type x Group | 0.449 | 1 | 534 | 0.503 |
|  | Trial order x mental age | 0.220 | 3 | 529 | 0.883 |
|  | Trial order x Group | 0.088 | 3 | 521 | 0.967 |
|  | Group x mental age | 0.700 | 1 | 533 | 0.403 |

**Table S6. Study 2 – Test phase**

The *Choice* (binary) variable was analysed by generalized linear mixed-effect models (GLMMs) with binomial distribution. Incuded factors: Condition (factor with two levels), Permanent Label type (factor with two levels), Group (factor with two levels), Trial order (factor with four levels). The model included the mental age as covariant, and the subject’s ID as random intercept. The table shows all the main and interaction effects along with their significance in the model (in case of nonsignificant effects it shows the p value before the elimination). Asterisks represent significant values (∗ p < 0.05, ∗∗ p < 0.01)

| **GLMM** | **Variable** | **F** | **df1** | **df2** | **p** |
| --- | --- | --- | --- | --- | --- |
| Main effects | Condition | 92.084 | 1 | 516 | < 0.001** |
|  | Permanent Label type | 3.285 | 1 | 516 | 0.070 |
|  | Group | 1.520 | 1 | 516 | 0.218 |
|  | Trial order | 3.655 | 3 | 516 | 0.012* |
|  | mental age | 0.006 | 1 | 516 | 0.939 |
| Interaction effects | Condition x Permanent Label type | 8.629 | 1 | 516 | 0.003** |
|  | Condition x Trial order | 0.289 | 3 | 502 | 0.833 |
|  | Condition x mental age | 0.025 | 1 | 501 | 0.874 |
|  | Condition x Group | 0.291 | 1 | 505 | 0.590 |
|  | Permanent Label type x Trial order | 1.316 | 3 | 506 | 0.268 |
|  | Permanent Label type x mental age | 5.837 | 1 | 516 | 0.016* |
|  | Permanent Label type x Group | 20.344 | 1 | 516 | < 0.001** |
|  | Trial order x mental age | 1.414 | 3 | 512 | 0.238 |
|  | Trial order x Group | 1.743 | 3 | 509 | 0.157 |
|  | Group x mental age | 3.748 | 1 | 515 | 0.053 |

**Effect sizes (TableS5-S7)**


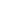


**Table S7. Study 1 – Prototype learning phase**

Performance: Parameter estimates with standard error (SE) between levels of test variables and statistical significance are given from the binomial GLMM for the main effects and interactions that proved to be significant in the model. The initial model included the age in months as covariant, Sex, Condition, Permanent Label type as factors and all the two way interactions. Asterisks represent significant values (∗ p < 0.05, ∗∗ p < 0.01).

| **Effects** | **Parameter Estimate ± SE** | **t** | **Odds ratio** | **p** | **95% Confidence interval** | |
| --- | --- | --- | --- | --- | --- | --- |
|  |  |  |  |  | **Lower** | **Upper** |
| Intercept | -0.624 ± 0.544 | -1.146 | 0.535 | 0.252 | -1.695 | 0.441 |
| age in months | -0.009 ± 0.008 | -1.108 | 0.991 | 0.268 | -0.025 | 0.007 |
| Condition (human figure -> dog figure) | 1.823 ± 0.736 | 2.476 | 6.190 | 0.013* | 0.381 | 3.270 |
| Age in months x Condition | -0.029 ± 0.011 | -2.520 | 0.971 | 0.012* | -0.051 | -0.006 |

**Table S8. Study 1 – Test phase**

Choice: The categorization responses (transient vs permanent: binary response variable) in Study 1 of N = 62 children. Chidren were repeatedly tested in two test conditions: Human figure vs Dog figure condition. The categorization was based on visualy detectable cues, Label types (skin/breed vs hair/fur). Parameter estimates with standard error (SE) between levels of test variables and statistical significance are given from the binomial GLMM for the main effects and interactions that proved to be significant in the model. The initial model included the age in months as covariant, Sex, Condition, Permanent Label type as factors and all the two way interactions. Asterisks represent significant values (∗ p < 0.05, ∗∗ p < 0.01).

| **Effects** | **Parameter Estimate ± SE** | **t** | **Odds ratio** | **p** | **95% Confidence interval** | |
| --- | --- | --- | --- | --- | --- | --- |
|  |  |  |  |  | **Lower** | **Upper** |
| Intercept | -2.08 ± 1.668 | -1.247 | 0.125 | 0.213 | 0.005 | 3.303 |
| Sex (female -> male) | 4.426 ± 1.824 | 2.426 | 83.62 | 0.015* | 2.329 | 3002.350 |
| age in months | 0.034 ± 0.025 | 1.354 | 1.035 | 0.176 | 0.985 | 1.087 |
| Condition (human figure -> dog figure) | -2.045 ± 0.237 | -8.624 | 0.129 | <0.01** | 0.081 | 0.206 |
| Trial order (4 -> 1) | 2.996 ± 1.761 | 1.701 | 19.999 | 0.089 | 0.630 | 634.346 |
| Trial order (4 -> 2) | 2.011 ± 1.768 | 1.137 | 7.469 | 0.256 | 0.232 | 240.243 |
| Trial order (4 -> 3) | -2.862 ± 1.791 | -1.597 | 0.057 | 0.111 | 0.002 | 1.926 |
| Permanent Label Type (hair/fur-> skin/breed) | 0.495 ± 0.439 | 1.127 | 1.641 | 0.260 | 0.693 | 3.888 |
| age in months * Sex = male | -0.057 ± 0.027 | -2.100 | 0.944 | 0.036* | 0.895 | 0.996 |
| age in months * Trial order = 1 | -0.038 ± 0.025 | -1.489 | 0.962 | 0.137 | 0.915 | 1.012 |
| age in months * Trial order = 2 | -0.036 ± 0.026 | -1.355 | 0.965 | 0.176 | 0.916 | 1.016 |
| age in months * Trial order = 3 | 0.049 ± 0.027 | 1.788 | 1.050 | 0.074 | 0.995 | 1.108 |
| Condition* Permanent Label Type | -0.914 ± 0.370 | -2.471 | 0.401 | 0.014* | 0.194 | 0.829 |
| Permanent Label Type (skin/breed) * Trial order = 1 | -1.699 ± 0.579 | -2.933 | 0.183 | 0.003** | 0.059 | 0.570 |
| Permanent Label Type (skin/breed) * Trial order = 2 | -0.501 ± 0.615 | -0.815 | 0.606 | 0.415 | 0.181 | 2.026 |
| Permanent Label Type (skin/breed) * Trial order = 3 | -0.334 ± 0.583 | -0.572 | 0.716 | 0.567 | 0.228 | 2.251 |

**Table S9. Study 2 – Test phase**

Choice: The categorization responses (transient vs permanent: binary response variable) in Study 2 of children assigned either to the experimental group (N=18 children with a diagnosis of autism spectrum disorder), or to the control group (N=17 neurotypical children). Children’s responeses were analysed using binomial generalized linear mixed models (GLMM, SPSS) with Condition (factor with two levels), Group (factor with two levels), Permanent Label Type (factor with two levels), Trial order (factor with four levels) and mental age (covariant) as fixed effects and ID as a random term. The table showes the Parameter estimates with standard error (SE) between levels of test variables and statistical significance for the main effects and the significant two way interaction effects.

| **Effects** | **Parameter Estimate ± SE** | **t** | **Odds ratio** | **p** | **95% Confidence interval** | |
| --- | --- | --- | --- | --- | --- | --- |
|  |  |  |  |  | **Lower** | **Upper** |
| Intercept | -1.266 ± 1.375 | -0.920 | 0.282 | 0.358 | 0.019 | 4.204 |
| mental age | 0.202 ± 0.228 | 0.884 | 1.224 | 0.377 | 0.782 | 1.915 |
| Condition (human figure -> dog figure) | -2.247 ± 0.361 | -6.221 | 0.106 | <0.01** | 0.052 | 0.215 |
| Permanent Label Type (hair/fur -> skin/breed) | 4.275 ± 1.147 | 3.727 | 71.880 | <0.01** | 7.550 | 685.002 |
| Trial order (4 -> 1) | -0.030 ± 0.390 | -0.078 | 0.970 | 0.938 | 0.451 | 2.089 |
| Trial order (4 -> 2) | 0.080 ± 0.454 | 0.176 | 1.083 | 0.860 | 0.444 | 2.644 |
| Trial order (4 -> 3) | 1.143 ± 0.430 | 2.652 | 3.136 | 0.008** | 1.345 | 7.312 |
| Group (Experimental -> Control) | 0.749 ± 0.669 | 1.118 | 2.115 | 0.264 | 0.567 | 7.875 |
| mental_age* Permanent Label Type | -0.437 ± 0.180 | -2.416 | 0.646 | 0.016* | 0.453 | 0.922 |
| Condition * Permanent Label Type | -1.769 ± 0.602 | -2.938 | 0.171 | 0.003** | 0.052 | 0.557 |
| Permanent Label Type *Group | -3.062 ± 0.678 | -4.510 | 0.047 | <0.01** | 0.012 | 0.178 |

**Post hoc analyses (TableS10-S12)**

**Table S10. Study 1 – Condition x Label type**

Differences in the use of Permanent Label type during the categorization task in each condition. Pairwise comparisons using estimated means-contrasts. Children were more likely to categorize dogs (dog figure condition) based on the permanent trait if that was indicated by breed than by fur colour.

| **Condition** | **Permanent Label type**  **Pairwise Contrasts** | **Contrast Estimate** | **SE** | **t** | **df** | **p** | **95% Confidence interval** | |
| --- | --- | --- | --- | --- | --- | --- | --- | --- |
|  |  |  |  |  |  |  | **Lower** | **Upper** |
| Dog figure | skin/breed - hair/fur | -0.116 | 0.034 | -3.475 | 891 | 0.001** | -0.182 | 0.050 |
| Human figure | skin/breed - hair/fur | -0.032 | 0.053 | -0.607 | 891 | 0.544 | -0.135 | 0.072 |

Table S9. Study 1 – Permanent Label Type x Set order

Between-Trial order differences in the mode of categorization (tranzient vs permanent) based on different Permanent Label types (skin/breed vs hair/fur). Pairwise comparisons using estimated means-contrasts. Children were more likely to categorize based on the permanent traits using the skin/breed label if that appeared at the beginning of the trials.

| **Label_type** | **Trial order Pairwise Contrasts** | **Contrast Estimate** | **SE** | **t** | **df** | **p** | **95% Confidence interval** | |
| --- | --- | --- | --- | --- | --- | --- | --- | --- |
|  |  |  |  |  |  |  | **Lower** | **Upper** |
| skin/breed | 1 - 2 | -0.057 | 0.058 | -0.974 | 891 | 0.661 | -0.187 | 0.074 |
|  | 1 - 3 | -0.240 | 0.085 | -2.836 | 891 | 0.028* | -0.464 | -0.017 |
|  | 1 - 4 | -0.231 | 0.081 | -2.841 | 891 | 0.028* | -0.445 | -0.016 |
|  | 2 - 3 | -0.183 | 0.085 | -2.171 | 891 | 0.121 | -0.395 | 0.028 |
|  | 2 - 4 | -0.174 | 0.087 | -2.008 | 891 | 0.135 | -0.382 | 0.034 |
|  | 3 - 4 | 0.009 | 0.101 | 0.093 | 891 | 0.926 | -0.189 | 0.208 |
| hair/fur | 1 - 2 | 0.190 | 0.082 | 2.311 | 891 | 0.126 | -0.027 | 0.407 |
|  | 1 - 3 | 0.020 | 0.092 | 0.220 | 891 | 0.839 | -0.161 | 0.202 |
|  | 1 - 4 | 0.111 | 0.083 | 1.339 | 891 | 0.724 | -0.096 | 0.318 |
|  | 2 - 3 | -0.169 | 0.082 | -2.059 | 891 | 0.199 | -0.382 | 0.043 |
|  | 2 - 4 | -0.079 | 0.083 | -0.953 | 891 | 0.839 | -0.271 | 0.113 |
|  | 3 - 4 | 0.091 | 0.084 | 1.082 | 891 | 0.839 | -0.110 | 0.292 |

**Table S11. Study 2 – Group x Permanent Label type**

Differences in the use of Permanent Label type during the categorization task in each group. Pairwise comparisons using estimated means-contrasts. Children in NT group were more likely to categorize based on permanent traits using skin/breed as Permanent label type, while the opposite pattern was observed in the ASD group.

| **Group** | **Permanent_label_type Pairwise Contrasts** | **Contrast Estimate** | **Std. Error** | **t** | **df** | **p** | **95% Confidence interval** | |
| --- | --- | --- | --- | --- | --- | --- | --- | --- |
|  |  |  |  |  |  |  | **Lower** | **Upper** |
| NT | skin/breed - hair/fur | -0.349 | 0.101 | -3.446 | 516 | 0.001** | -0.549 | -0.150 |
| ASD | skin/breed - hair/fur | 0.232 | 0.092 | 2.519 | 516 | 0.012* | 0.051 | 0.413 |

**Table S12. Study 2 – Condition x Permanent Label type**

Differences in the use of Permanent Label type during the categorization task in each condition. Pairwise comparisons using estimated means-contrasts. Children in dog figure condition group were more likely to categorize based on permanent traits using skin/breed as Permanent label type, while the oppesite pattern was observed in the human figure condition.

| **Condition** | **Permanent_label_type Pairwise Contrasts** | **Contrast Estimate** | **Std. Error** | **t** | **df** | **p** | **95% Confidence interval** | |
| --- | --- | --- | --- | --- | --- | --- | --- | --- |
|  |  |  |  |  |  |  | **Lower** | **Upper** |
| dog | skin/breed - hair/fur | -0.109 | 0.046 | -2.378 | 516 | 0.018* | -0.198 | -0.018 |
| human | skin/breed - hair/fur | 0.079 | 0.078 | 1.010 | 516 | 0.313 | -0.074 | 0.232 |

**Supplementary figure**

**Figure S1**. The Sex x Age in months interaction effect for the regions of significance indicated by the Johnson-Neyman technique.


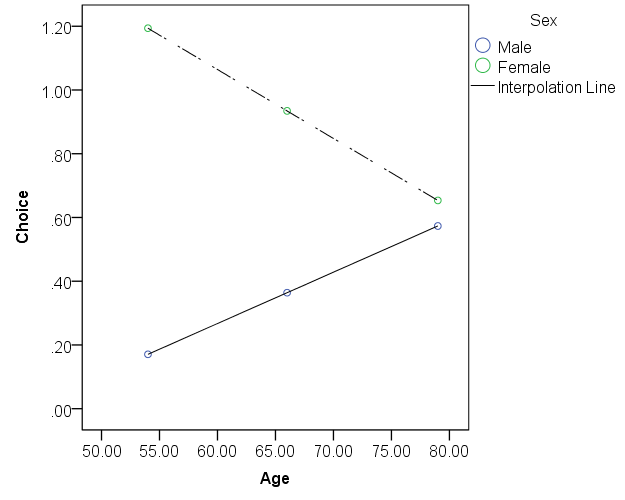

Supplement: Supplementary file 1 — Supplementary Information [file 41598_2021_85924_MOESM1_ESM.docx]
